# Introducing an *Arabidopsis thaliana* Thylakoid Thiol/Disulfide-Modulating Protein Into *Synechocystis* Increases the Efficiency of Photosystem II Photochemistry

**DOI:** 10.3389/fpls.2019.01284

**Published:** 2019-10-16

**Authors:** Ryan L. Wessendorf, Yan Lu

**Affiliations:** Department of Biological Sciences, Western Michigan University, Kalamazoo, MI, United States

**Keywords:** photosynthesis, Photosystem II, thylakoid thiol/disulfide-modulating protein, PSII photochemical efficiency, *Arabidopsis thaliana*, *Synechocystis*

## Abstract

Photosynthetic species are subjected to a variety of environmental stresses, including suboptimal irradiance. In oxygenic photosynthetic organisms, a major effect of high light exposure is damage to the Photosystem II (PSII) reaction-center protein D1. This process even happens under low or moderate light. To cope with photodamage to D1, photosynthetic organisms evolved an intricate PSII repair and reassembly cycle, which requires the participation of different auxiliary proteins, including thiol/disulfide-modulating proteins. Most of these auxiliary proteins exist ubiquitously in oxygenic photosynthetic organisms. Due to differences in mobility and environmental conditions, land plants are subject to more extensive high light stress than algae and cyanobacteria. Therefore, land plants evolved additional thiol/disulfide-modulating proteins, such as Low Quantum Yield of PSII 1 (LQY1), to aid in the repair and reassembly cycle of PSII. In this study, we introduced an *Arabidopsis thaliana* homolog of LQY1 (AtLQY1) into the cyanobacterium *Synechocystis* sp. PCC6803 and performed a series of biochemical and physiological assays on AtLQY1-expressing *Synechocystis*. At a moderate growth light intensity (50 µmol photons m^-2^ s^-1^), AtLQY1-expressing *Synechocystis* was found to have significantly higher *F* *_v_* */F* *_m_*, and lower nonphotochemical quenching and reactive oxygen species levels than the empty-vector control, which is opposite from the loss-of-function *Atlqy1* mutant phenotype. Light response curve analysis of PSII operating efficiency and electron transport rate showed that AtLQY1-expressing *Synechocystis* also outperform the empty-vector control under higher light intensities. The increases in *F* *_v_* */F* *_m_*, PSII operating efficiency, and PSII electron transport rate in AtLQY1-expressing *Synechocystis* under such growth conditions most likely come from an increased amount of PSII, because the level of D1 protein was found to be higher in AtLQY1-expressing *Synechocystis*. These results suggest that introducing AtLQY1 is beneficial to *Synechocystis*.

## Introduction

Photosynthesis provides chemical energy for nearly all life forms on earth. In oxygenic photosynthesis, which occurs in cyanobacteria, algae, and land plants, photosynthetic electron transport and ATP synthesis requires Photosystem II (PSII), cytochrome *b*_6_ *f*, Photosystem I (PSI), ATP synthase, as well as mobile electron carriers such as plastoquinone and plastocyanin (Allen et al., 2011; Nickelsen and Rengstl, 2013). During the evolution from cyanobacteria to land plants, core components of the photosynthetic apparatus have been conserved (Allen et al., 2011; Nickelsen and Rengstl, 2013). For example, the core subunits of PSII in cyanobacteria, algae, and land plants are similar except for the composition of light harvesting complexes (LHCs) and oxygenic evolving complexes (OECs) (Hankamer et al., 2001; Allen et al., 2011; Nickelsen and Rengstl, 2013). The LHCs of cyanobacterial and red algal PSII supercomplexes are termed phycobilisomes and consist of three types of phycobiliproteins: allophycocyanin (APC), phycocyanin (PC), and phycoerythrin (PE) (Stadnichuk et al., 2015). These phycobiliproteins contain one or multiple cysteine residues. Phycobilisome chromophores allophycocyanobilin (APCB), phycocyanobilin (PCB), and phycoerythrobilin (PEB) are covalently attached to APC, PC, and PE subunits, respectively, via thioether bonds to conserved cysteine residues (Zhao et al., 2006). The LHCs of PSII (i.e., LHCII) in land plants consist of trimeric antenna proteins LHCB1 (LHCB stands for PSII light-harvesting chlorophyll *a*/*b*-binding protein), LHCB2, and LHCB3, as well as monomeric antenna proteins LHCB4, LHCB5, and LHCB6 (Ballottari et al., 2012). The OEC in cyanobacteria consists of five extrinsic proteins: PsbO, PsbP-like, PsbQ-like, PsbU, and PsbV (Hankamer et al., 2001; Thornton et al., 2004; Bricker et al., 2012). The OEC in green algae and land plants only has three proteins: PsbO, PsbP and PsbQ (Hankamer et al., 2001; Thornton et al., 2004; Bricker et al., 2012). PsbU and PsbV were lost during the evolution of green algae and land plants (Thornton et al., 2004).

Photosynthetic species are subject to a wide range of environmental stresses, such as drought, flood, high salinity, extreme temperature, and to the main interest of this work, suboptimal light intensities. In oxygenic photosynthetic organisms, a major consequence from high light exposure is damage to PSII core proteins, especially PSII reaction-center protein D1 (Demmig-Adams and Adams, 1992; Aro et al., 1993). To minimize photodamage and photoinhibition, photosynthetic organisms have evolved photoprotection and repair strategies, such as increased thermal dissipation [e.g., non-photochemical quenching (NPQ)] at the antennae level and accelerated PSII repair at the reaction-center level (Liu et al., 2019). NPQ mechanisms differ among cyanobacteria, algae, and land plants (Gorbunov et al., 2011; Kirilovsky and Kerfeld, 2016; Misumi et al., 2016). In land plants, the major NPQ component is energy-dependent quenching mediated by the xanthophyll cycle (Demmig-Adams and Adams, 1996). Algae have diverse antennae systems, thus different algal species have different mechanisms of energy-dependent quenching (Goss and Lepetit, 2015). Cyanobacteria do not have the xanthophyll cycle (Demmig-Adams et al., 1990; Campbell et al., 1998). NPQ in cyanobacteria is mediated by the orange carotenoid protein (OCP), a soluble stromal protein that acts as a homodimer (Kerfeld et al., 2003; Wilson et al., 2010; Gupta et al., 2019). Strong white (or blue-green) light was found to cause OCP photoactivation and binding to phycobilisomes, which induces OCP-mediated NPQ (Kirilovsky, 2007; Kirilovsky, 2015; Kirilovsky and Kerfeld, 2016). The N-terminal effector domain and the C-terminal regulator domain of monomeric OCP contain two and one cysteine residues, respectively; and the cysteine residue in the C-terminal domain was found to be critical for dimerization and activation of OCP (Moldenhauer et al., 2017; Muzzopappa et al., 2017). Although NPQ avoids photodamage and photoinhibition, it occurs at the cost of reduced photosynthetic efficiency. Thus, down regulation and fine tuning of NPQ is a target of improving photosynthetic efficiency (Berteotti et al., 2016; Kromdijk et al., 2016; Perozeni et al., 2019).

Unlike NPQ, major steps of the damage, repair, and reassembly cycle of PSII are highly conserved among cyanobacteria, algae, and land plants (Mulo et al., 2008; Nixon et al., 2010; Nickelsen and Rengstl, 2013; Nickelsen and Zerges, 2013; Rast et al., 2015; Lu, 2016). This process occurs under low or moderate light intensity as well, although at a slower speed (Aro et al., 1993; Foyer and Shigeoka, 2011). In brief, the inactive PSII complexes with photodamaged D1 are partially dissembled to facilitate the degradation of photodamaged D1 and the co-translational insertion of the nascent D1 protein. After the replacement of D1, the PSII complexes are re-assembled to restore function. It was proposed that folding, disassembly, and re-assembly of PSII proteins and complexes may involve transient formation and breakage of inter- and/or intra-molecular disulfide bonds between cysteine residues (Zhang and Aro, 2002; Shimada et al., 2007; Karamoko et al., 2011; Lu et al., 2011). Interestingly, a number of PSII proteins contain cysteine residues, including hydrophobic PSII core subunits D1, D2, CP43, and CP47, as well as hydrophilic OEC subunits PsbO, PsbP, and PsbQ (Shimada et al., 2007). Exploiting the PSII repair and reassembly cycle is another target of improving photosynthetic efficiency (Liu et al., 2019).

The elaborate PSII repair and reassembly cycle requires auxiliary proteins of different functions, such as D1 C-terminal processing, thiol/disulfide-modulating, peptidylprolyl isomeration, phosphorylation, and dephosphorylation (Mulo et al., 2008; Nixon et al., 2010; Nickelsen and Rengstl, 2013; Lu, 2016). Although some auxiliary proteins are unique to land plants, algae, or cyanobacteria, most auxiliary proteins exist ubiquitously in oxygenic photosynthetic organisms (Komenda et al., 2012; Nickelsen and Rengstl, 2013). One example of thiol/disulfide-modulating auxiliary proteins that exist ubiquitously in oxygenic phyotosynthetic organisms is Lumen Thiol Oxidoreductase 1 (LTO1). *Arabidopsis thaliana* LTO1 and its cyanobacterial homologs were found to catalyze disulfide bond formation in lumenal and lumen-exposed proteins, thus regulating PSII assembly and redox homeostasis (Singh et al., 2008a; Furt et al., 2010; Li et al., 2010; Feng et al., 2011; Karamoko et al., 2011; Lu et al., 2013). LTO1 contains an N-terminal vitamin K epoxide reductase (VKOR)-like domain with five transmembrane segments and a C-terminal thioredoxin-like domain. The thioredoxin-like domain in LTO1 was found to interact with lumen-exposed PSII OEC proteins PsbO1 and PsbO2 and a thylakoid lumenal peptidyl-prolyl isomerase FKBP13 [FK506 (tacrolimus)-binding protein 13] (Karamoko et al., 2011; Lu et al., 2013). The LTO1 homolog in the green alga *Chlamodonas reinhardtii*, which is encoded by Cre12.g493150, has not been characterized (Nickelsen and Rengstl, 2013).

In comparison to algae and cyanobacteria, land plants are subject to more extensive high light stress, due to the difference in mobility and environmental conditions (Lu, 2011; Nickelsen and Rengstl, 2013; Wessendof, 2017). Aquatic algae and cyanobacteria can vary their depth in lakes and oceans to avoid the damaging effects of exposure to high light, while land plants do not have the capability of escaping. Land plants evolved additional thiol/disulfide-modulating proteins, such as CYO1/SCO2 (Shiyou1/Snowy Cotyledon2) (Shimada et al., 2007; Albrecht et al., 2008; Muranaka et al., 2012; Tanz et al., 2012) and LQY1 (Low Quantum Yield of PSII 1) (Lu, 2011; Lu et al., 2011), to aid in the repair and reassembly cycle of PSII. LQY1 homologs are present in land plants (e.g., AtLQY1 in *Arabidopsis thaliana*) but are not found in the sequenced genomes of aquatic algae and cyanobacteria, suggesting that LQY1 may play a role in plant adaptations to life on land (Lu, 2011). AtLQY1 is a small thylakoid zinc-finger protein with four CXXCXGXG repeats and an N-terminal transmembrane domain anchoring the protein to the thylakoid membrane from the stromal side (Lu et al., 2011). Inductively coupled plasma-mass spectrometry analysis of affinity-purified recombinant AtLQY1 protein showed that each LQY1 peptide contains two zinc ions, coordinately by the cysteine residues in four CXXCXGXG repeats (Lu et al., 2011). The zinc-finger domain of AtLQY1 also demonstrated protein disulfide isomerase activity (i.e., thiol/disulfide-modulating activity) towards thiol/disulfide-containing protein substrates. Thus, LQY1 was proposed to participate in folding, disassembly, and/or assembly of cysteine-containing PSII subunits in land plants (Lu, 2016).

Loss-of-function *Atlqy1* mutants were more sensitive to light stress than the wild type, had higher *NPQ* values, and accumulated more reactive oxygen species (ROS) than the wild type after the high light treatment (Lu et al., 2011). Under elevated light conditions, the *Atlqy1* mutants had fewer PSII-LHCII supercomplexes and lower PSII maximum efficiency than the wild type. In line with these observations, AtLQY1 was found to be associated with the PSII core monomer and the CP43-less PSII monomer (a marker for ongoing PSII repair and reassembly; Boehm et al., 2012). The proportion of PSII monomer-associated AtLQY1 increased substantially after prolonged high light treatment. Furthermore, cysteine-containing PSII core subunits CP47 and C43 were found to co-immunoprecipitate with the anti-AtLQY1 antibody. Therefore, it was concluded that LQY1 may regulate PSII repair and reassembly by forming transient disulfide bonds with cysteine-containing PSII subunits and regulate redox homeostasis by reducing ROS accumulation (Lu, 2011; Lu et al., 2011).

In this study, we introduced AtLQY1 into the model cyanobacterium *Synechocystis* sp. PCC6803 (*Synechocystis*), performed a series of biochemical and physiological assays on AtLQY1-expressing *Synechocystis*, and compared with the empty-vector control. We are particularly interested in knowing whether AtLQY1 expression improves PSII photochemical efficiency in *Synechocystis*.

## Materials and Methods

### Introducing AtLQY1 Into *Synechocystis*

The coding sequence of full-length AtLQY1 (AtLQY1^1-154^) (Lu et al., 2011) was amplified by PCR using primers Nde1_LQY1_F and Hpa1_LQY1_R (**Supplementary Table S1**). The resulting PCR product was AT-cloned into the pGEM-T Easy Vector and sequenced with primers M13_Forward and_M13 Reverse (**Supplementary Table S1**) to confirm the absence of PCR errors. Nde1/Hpa1-digested *AtLQY1* fragment was subcloned into the *Synechocystis* expression vector pSL2035. The resulting construct was sequenced to confirm correct insertion and absence of errors. Thirty milliliters of wild-type *Synechocystis* was grown continuously at 50 µmol photons m^-2^ s^-1^ to an optical density of 0.60 at 730 nm (i.e., OD_730_ = 0.60), in a 125-ml Erlenmeyer flask containing BG-11 liquid medium. To minimize cell damage, *Synechocystis* cells were gently harvested via centrifugation at 2,760 g for 10 min at 4°C. The cell pellet was washed twice with 5 ml of fresh BG-11 medium. The washed cell pellet was resuspended in 1.5 ml of fresh BG-11 medium. pSL2035-AtLQY1 and the empty pSL2035 vector constructs were mixed with *Synechocystis* cell suspensions to the concentration of 1 µg/ml in a 300-µl final volume. Cells were incubated at 28°C at 50 µmol photons m^-2^ s^-1^ for 5 h, and were gently inverted every hour. The resulting cultures were plated on a piece of autoclaved filter paper on BG-11 solid medium supplemented with 25 µg/ml kanamycin and examined for colonies in two weeks. Candidate transformants (colonies) were genotyped with the Nde1_LQY1_F forward primer and the psbA1d_100_down_R and psbA1d_200_down_R reverse primers (**Supplementary Table S1**) to ensure proper insertion of exogenous DNA. Confirmed transformants were streaked to fresh BG-11 plates supplemented with 50 µg/ml kanamycin to ensure a more homoplasmidic state.

### Culture Growth Conditions

*Synechocystis* cultures transformed with pSL2035-AtLQY1 or the empty pSL2035 vector were grown in BG-11 liquid medium or on BG-11 plates supplemented with 25 µg/ml kanamycin (Varman, 2010; Eaton-Rye, 2011; Ermakova et al., 2016). All liquid cultures (30 ml) were grown in 125-ml Erlenmeyer flasks with a culture depth of 1 cm on a VWR mini shaker set at 140 rpm in a growth chamber (Percival). The temperature was set to 28°C and the light intensity set to 25 or 50 µmol photons m^-2^ s^-1^ at the surface of the flasks. The Percival reach-in chamber used in this study was equipped with a mixed array of fluorescent and incandescent lamps designed to produce a broader spectral range: eight 25-W T8 standard fluorescent tube light bulbs (Philips F25TB/TLB841) and four 100-W incandescent light bulbs (Westinghouse Commercial Service). For 25 and 50 µmol photons m^-2^ s^-1^ light intensities, six 25-W T8 standard fluorescent tube light bulbs and two 100-W incandescent light bulbs were used and the difference between two light intensities was achieved by adjusting the distance between the shelf and the light fixture. To ensure uniform light quality and quantity, all bulbs were replaced before they reached the end of their lifetime. Solid cultures were grown on a growth station with similar growth conditions. Samples were collected from liquid cultures at the mid-log phase (OD_730_ = 0.50 to 0.70) for all downstream assays unless otherwise stated.

### Total Protein Extraction

Mid-log phase *Synechocystis* cultures (10 ml) were harvested via centrifugation at 3,220 g for 20 min at 4°C. The pellets were resuspended in 400 µl of lysis buffer (0.1 M NaOH, 0.025 M EDTA, 2% SDS, 1 mM DTT) and incubated at 60°C for 10 min. The lysed cells were neutralized with 10 µl of 4 M acetic acid, and mixed thoroughly by vortexing for 30 s. The resuspended samples were centrifuged at 14,000 g for 2 min, after which the supernatant was collected. The total protein concentration in the supernatant was determined with the DC protein assay kit (Bio-Rad). Protein samples were diluted into an equal final total protein concentration (4 µg/ml) and an appropriate volume of 5X loading buffer (0.025 M EDTA, 0.25 M Tris-HCl, pH = 6.8, 50% glycerol, 39 mM DTT, 0.05% bromophenolblue) was added. The resulting protein samples were used for SDS-Urea-PAGE and immunoblot analysis.

### SDS-Urea-PAGE and Immunoblot Analysis

Total protein samples were loaded on an equal culture OD_730_ basis and separated by SDS-Urea-PAGE (15% polyacrylamide, 6 M urea). Proteins were then transferred to a polyvinylidene difluoride membrane in a Trans-Blot electrophoresis transfer cell (Bio-Rad). The membrane was blocked in a blocking solution (5% nonfat dry milk, 0.1% Tween-20 in 1X Tris Buffered Saline), and then incubated in diluted antibody solutions (Lu et al., 2011). The anti-AtLQY1 antibody was made by Open Biosystems (Lu et al., 2011); the anti-PsaA, anti-D1 (C-terminus of D1), anti-D2, anti-APC, and anti-PC antibodies were purchased from Agrisera. Immunodetection was achieved with the SuperSignal west pico rabbit immunoglobulin G detecting kit (Thermo Fisher) and the Gel Logic 1500 Imaging System (Kodak), as described previously (Hackett et al., 2017).

### Chlorophyll *a* and Carotenoid Content Measurements

Chlorophyll (Chl) *a* and carotenoids are extracted as previously described (Zavřel et al., 2015). In brief, *Synechocystis* cultures (1 ml) were harvested at an OD_730_ of ∼0.7 via centrifugation at 15,000 g for 7 min at 4°C. The pellets were resuspended in 1 ml of pre-chilled (4°C) methanol. After 4-s vortexing to obtain homogenization, samples were incubated in the dark at 4°C for 20 min. To remove cell residues, samples were centrifuged at 15,000 g for 7 min at 4°C. The optical densities of the supernatants were measured at 470, 665, and 720 nm with a BioMate 3S spectrophotometer (Thermo Fisher). The Chl *a* content (µg/ml) was calculated as: 12.9447 * (OD *_665_* *– OD* *_720_* *)* (Ritchie, 2006); the carotenoid content (µg/ml) was calculated as: (1,000 * (OD *_470_* *– OD* *_720_* ) – 2.86 * Chl a [µ*g/ml])/ 221* (Wellburn, 1994). It should be noted that cyanobacteria such as *Synechocystis* do not produce Chl *b*.

### Phycobilisome Pigment Measurements

The contents of phycobilisome pigments APCB, PCB, and PEB were determined as previously described (Hsieh et al., 2014), with some modifications explained in von der Haar (2007). Pellets from Chl *a* and carotenoid extraction were washed twice with 1 ml of 6 mM EDTA (pH 8.0). Washed pellets were resuspended with 50 µl of 6 mM EDTA (pH 8.0) and 700 µg/ml lysozyme, and incubated at 37°C for one hour with one shake at 30 min. Next, 50 µl of 4 M sodium hydroxide was added to each sample and all the samples were incubated at room temperature for 5 min. After re-pelleting, the supernatants were transferred to new centrifuge tubes and 100 µl of 1.5 M TRIS-HCl (pH 6.8) was added as a neutralizer to re-establish pigmentation of the samples. Samples were loaded on a 96-well flat bottom plate (Greiner). The optical densities of the samples were measured at 562 nm, 615 nm, and 652 nm on an Epoch microplate spectrophotometer (BioTek) equipped with the Gen5 software. APCB was calculated as: *(OD* *_652_* – 0.208 * OD *_615_* *) / 5.09*, PCB was calculated as: *(OD* *_615_* – 0.474 * OD *_652_* *) / 5.34* and PEB was calculated as: *(OD* *_652_* – 2.41 * PCB [µg/ml] – 0.849 * APCB [µg/ml]) / 9.62 (Hsieh et al., 2014).

### Measurements of Fluorescence Parameters

Measurements of minimal fluorescence (*F* *_o_*), maximal fluorescence (*F* *_m_*), variable fluorescence (*F* *_v_*), *F* *_v_* */F* *_m_* (a relative measure of PSII maximum photochemical efficiency), light response curves of PSII operating efficiency (*Φ* *_PSII_*), and electron transport rate (*ETR* *_PSII_*) in *Synechocystis* cultures were performed as described previously (Sauer et al., 2001; Barthel et al., 2013), with minor modifications. *Synechocystis* cultures (2 ml) were harvested at an OD_730_ of ∼0.7 and dark adapted for 5 min in the quartz cuvette of the DUAL-PAM-100 measuring system (Walz, Germany). Cultures were resuspended, exposed to a saturation pulse (2,000 µmol photons m^-2^ s^-1^) to determine *F* *_o_* and *F* *_m_* of dark-adapted cultures, and then illuminated for 30 s at the following light intensities: 0, 8, 13, 20, 46, 82, 105, 161, 236, and 422 µmol photons m^-2^ s^-1^. A saturation pulse (2,000 µmol photons m^-2^ s^-1^) was applied at the end of each 30-s illumination to determine fluorescence parameters of illuminated cultures. *F* *_v_* and *F* *_v_* */F* *_m_* of dark-adapted cultures were calculated using the following equations: *F* *_v_* = *F* *_m_* – *F* *_o_*; *F* *_v_* */F* *_m_* = (*F* *_m_* – *F* *_o_*)/*F* *_m_*. Φ*_PSII_* was calculated using the following equation: *Φ* *_PSII_* = (*F*_m_’ – *F*)/*F*_m_’ , where *F*_m_’ and *F* are maximal and current fluorescence. *ETR*_PSII_ was calculated as: *Φ* *_PSII_* * PAR * A *_culture_* * Fraction *_PSII_*, where PAR is incident photosynthetic active radiation, *A* *_culture_* is the ratio of incident photons absorbed by cultures (0.84) and *Fraction* *_PSII_* is the ratio of absorbed photons distributed to PSII. Depending on the quality and intensity of growth light, the PSI:PSII ratio in cyanobacteria varies between 5:1 and 2:1 (Shen et al., 1993; Murakami et al., 1997; Luimstra et al., 2018). Under a growth light of 10-65 µmol photons m^-2^ s^-1^ with a broader spectral range, the PSI:PSII ratio is ∼2.5:1 in *Synechocystis* (Fraser et al., 2013). Therefore, a *Fraction* *_PSII_* of 0.29 (i.e., 1/3.5) was used to calculate *ETR*_PSII_ in the empty-vector control grown at 25 µmol photons m^-2^ s^-1^. The *Fraction* *_PSII_* of AtLQY1-expressing *Synechocystis* grown at 25 and 50 µmol photons m^-2^ s^-1^ (0.34 and 0.27, respectively) and the empty-vector control grown at 50 µmol photons m^-2^ s^-1^ (0.25) was estimated from the abundance of the PsaA and D1 proteins.

### NPQ Measurements

NPQ was measured with a DUAL-PAM-100 measuring system (Waltz), as described previously (Gorbunov et al., 2011). Cultures were harvested at an OD_730_ of ∼0.7. After a 5-min dark adaption, cultures were resuspended, and a saturation pulse (2,000 µmol photons m^-2^ s^-1^) was applied to determine *F* *_o_* and *F* *_m_* of dark-adapted cultures. Cultures were pre-illuminated under a measuring light of 2 µmol photons m^-2^ s^-1^ for 3 min, with saturating pulses at 30-s intervals. After the 3-min pre-illumination, blue actinic light (422 µmol photons m^-2^ s^-1^) was applied for 8 min with saturating pulses at 20-s intervals. Recovery was monitored under a measuring light of 2 µmol photons m^-2^ s^-1^ for 18 min, with exponentially increasing intervals between saturating pulses. *NPQ* was calculated using the following equation: (*F*_m_ – *F*_m_’)/*F*_m_’, where *F*_m_ is maximal fluorescence of dark-adapted cultures and *F*_m_’ is maximal fluorescence near the end of blue actinic illumination.

### High Light Treatment

High light treatment was performed according to Singh et al. (2008b) with some modifications (Singh et al., 2008b). *Synechocystis* cultures used for high light experiments were grown in the Percival growth chamber at 50 µmol photons m^-2^ s^-1^ till mid-log phase (OD_730_ of ∼0.7). Prior to the high light treatment, an aliquot (2 ml) of cultures was harvested and dark-adapted for chlorophyll fluorescent measurements of *F* *_v_* */F* *_m_*. The remaining cultures were left in the growth chamber and the light intensity was increased to a moderately high intensity of 250 µmol photons m^-2^ s^-1^, while all other conditions in the growth chamber remained constant. After the 90-min high light treatment, another aliquot (2 ml) of cultures was harvested and dark-adapted for *F* *_v_* */F* *_m_* measurements.

### ROS Measurements

The total amount of ROS was determined by using the ROS indicator 2’,7’-dichlorodihydro fluorescin diacetate (DCHF-DA), as previously described (Singh and Montgomery, 2012; Lea-Smith et al., 2013). The DCHF-DA probe is cell permeable and becomes highly fluorescent when oxidized to dichloroflurescin (DCF) by intracellular ROS, such as H_2_O_2_, hydroxyl and peroxyl radicals, and peeroxynitrite (Kalyanaraman et al., 2012; Lea-Smith et al., 2013). *Synechocystis* cultures (3 ml) were harvested at an OD_730_ of ∼0.7, twice washed and resuspended with 1X TES buffer (pH 8.2), to reduce background noise from BG-11 media. DCHF-DA was dissolved in N,N-dimethylformamide and added to appropriate cell samples at a final concentration of 50 µM. After a 30-min dark incubation, fluorescence in all samples was measured on a fluorescence spectrophotometer (Varian Cary Eclipse) with an excitation wavelength of 485 nm and emission wavelengths from 500 to 600 nm in a 96-well plate. Measurements were taken at four wavelengths (520 nm, 525 nm, 530 nm, and 535 nm) and were normalized to OD_730_. TES buffer containing the DCHF-DA probe was used as the negative control; the positive control consisted of cells treated with 100 µM methyl viologen (Thomas et al., 1998). Fluorescence in cell samples not treated with DCHF-DA was subtracted from cells samples treated with DCHF-DA.

### Transmission Electron Microscopy

*Synechocystis* cells were prepared for transmission electron microscopy (TEM) analysis as described in Tsang et al. (2013), with some modifications. *Synechocystis* cultures (30 ml) were harvested at an OD_730_ of ∼0.7. Cells were gently pelleted by centrifugation at 2,000 g for 10 min at 4°C. The supernatant was removed and the cells were re-suspended in a fixative solution (formaldehyde/glutaraldehyde, 2.5% each in 0.1 M sodium cacodylate buffer, pH 7.4). After primary fixation, samples were washed with 0.1 M cacodylate buffer and postfixed with 1% osmium tetroxide in 0.1 M cacodylate buffer, dehydrated in a gradient series of acetone and infiltrated and embedded in Spurr’s resin. 70-nm thin sections were obtained with a Power Tome Ultramicrotome (RMC Boeckeler Instruments) and post-stained with uranyl acetate and lead citrate. Images were taken with JEOL 100CX Transmission Electron Microscope (Japan Electron Optics Laboratory, Japan) at an accelerating voltage of 100 kV at the Michigan State University Center for Advance Microscopy.

Thylakoid membrane spacing distances were measured from the TEM images and calibrated with the pixel size of TEM images as described previously (Schindelin et al., 2012; Liberton et al., 2013; Majumder et al., 2017). Approximately three measurements were taken on each cell for ten cells per cell type per growth condition.

### Accession Numbers

Sequences data of related genes/proteins can be found in the GenBank/EMBL databases under the following accession numbers: AtLQY1, At1g75690.

## Results

### Expression of AtLQY1 in *Synechocystis*

**Figure 1:**
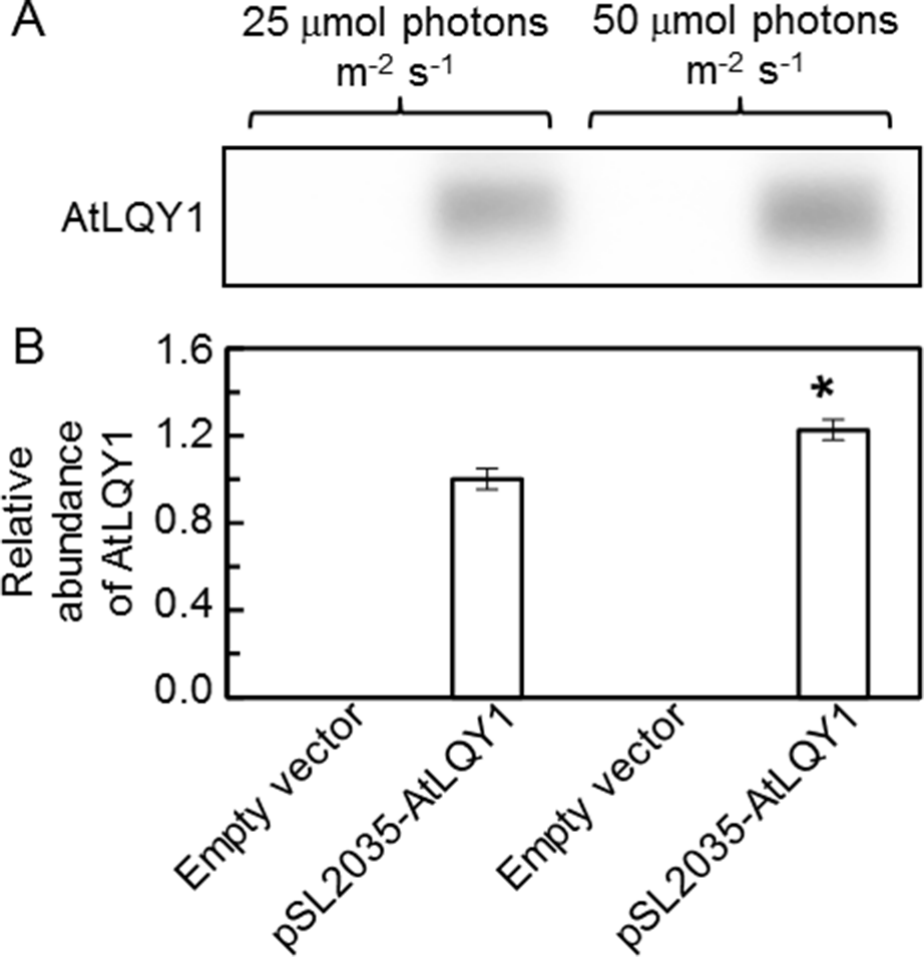
Immunoblot analysis of AtLQY1 expression in *Synechocystis* cultures grown at 25 and 50 µmol photons m^-2^ s^-1^. **(A)** Representative immunoblot of AtLQY1. Total protein samples were loaded on an equal culture OD_730_ basis. **(B)** Relative abundance of AtLQY1. Data are presented as mean ± SE (n = 4 independent biological replicates). The asterisks indicate significant differences between AtLQY1–expressing *Synechocystis* grown at 25 and 50 µmol photons m^-2^ s^-1^ (Student’s *t*-test; *, *p* < 0.05).

To introduce AtLQY1 into *Synechocystis*, the coding region of full-length AtLQY1 (Lu, 2011; Lu et al., 2011) was subcloned into the *Synechocystis* expression vector pSL2035. The pSL2035 vector is designed to integrate a gene of interest into the *psbA1* gene site in the *Synechocystis* genome, using homologous double recombination. The *psbA1* gene site in wild-type *Synechocystis* is silent under most conditions (Varman, 2010). Expression of the gene of interest is controlled by the *PsbA2* promoter (Varman, 2010). The integration of the *PsbA2* promoter and the gene of interest to the *psbA1* gene site allows overexpression of the gene of interest without causing untargeted physiological effects. pSL2035-AtLQY1 and the empty pSL2035 vector were transformed into wild-type *Synechocystis*, as described previously (Varman, 2010; Varman et al., 2013). Candidate transformants were genotyped to confirm successful transformation and were streaked to fresh BG-11 plates supplemented with 50 µg/ml kanamycin to achieve homoplasmidity. Before *Synechocystis* transformants were used in detailed phenotypic characterization, we tested the expression level of AtLQY1 with SDS-Urea-PAGE and immunoblot analysis. The result confirmed successful expression of AtLQY1 in *Synechocystis* transformants (**Figure 1A**). The AtLQY1 protein level in *Synechocystis* transformants grown at 50 µmol photons m^-2^ s^-1^ was significantly higher than that in *Synechocystis* transformants grown at 25 µmol photons m^-2^ s^-1^ (**Figure 1B**, **Supplementary Table S2**). This observation is consistent with the use of the light-inducible *psbA2* promoter to express the exogenous *AtLQY1* gene (Mohamed and Jansson, 1989; Lindberg et al., 2010).

### Cell Counts in AtLQY1-Expressing *Synechocystis*

The cell count in AtLQY1-expressing *Synechocystis* and the empty-vector control was comparable at both growth light intensities (**Table 1**). As the growth light intensity increased from 25 to 50 µmol photons m^-2^ s^-1^, the cell count in both AtLQY1-expressing *Synechocystis* and the empty-vector control doubled (**Table 1**).

**Table 1:** Cell counts, pigment contents and chlorophyll fluorescence parameters.

Parameter	25 µmol photons m^-2^ s^-1^		50 µmol photons m^-2^ s^-1^	
	Empty vector	pSL2035-AtLQY1	Empty vector	pSL2035-AtLQY1
Cell count (10^6^/ml)	1.40 ± 0.05^B^	1.41 ± 0.13^B^	2.76 ± 0.08^A^	2.56 ± 0.07^A^
Chl *a* (µg/ml)	4.14 ± 0.24^A^	4.00 ± 0.25^AB^	3.31 ± 0.32^B^	3.69 ± 0.25^AB^
Carotenoid (µg/ml)	1.79 ± 0.12^A^	1.72 ± 0.12^A^	1.74 ± 0.21^A^	2.05 ± 0.11^A^
Chl *a*/carotenoid	2.32 ± 0.06^A^	2.33 ± 0.02^A^	1.92 ± 0.06^B^	1.79 ± 0.03^B^
APCB (µg/ml)	21.65 ± 1.08^A^	16.26 ± 1.81^B^	9.05 ± 0.96^C^	9.54 ± 0.92^C^
PCB (µg/ml)	58.25 ± 1.63^A^	43.18 ± 2.70^B^	50.65 ± 3.93^AB^	49.84 ± 3.37^AB^
PEB (µg/ml)	3.66 ± 0.35^A^	2.82 ± 0.64^A^	Negligible	Negligible
PCB/APCB	2.70 ± 0.06^B^	2.69 ± 0.19^B^	5.70 ± 0.47^A^	5.30 ± 0.39^A^
F_v_ /F_m_	0.261 ± 0.003^B^	0.259 ± 0.008^B^	0.281 ± 0.010^B^	0.344 ± 0.007^A^
F_o_	0.154 ± 0.007^B^	0.180 ± 0.013^A^	0.110 ± 0.003^C^	0.170 ± 0.003^AB^
F_m_	0.209 ± 0.010^B^	0.243 ± 0.019^AB^	0.153 ± 0.004^C^	0.260 ± 0.008^A^
F_v_	0.055 ± 0.003^BC^	0.063 ± 0.006^B^	0.043 ± 0.002^C^	0.090 ± 0.004^A^
NPQ	0.695 ± 0.017^A^	0.695 ± 0.020^A^	0.562 ± 0.023^B^	0.381 ± 0.010^C^

Measurements for cell counts, pigment contents, and fluorescence parameters were performed on Synechocystis cultures grown at 25 and 50 µmol photons m^-2^ s^-1^. Data are normalized to 1 ml of culture and are presented as mean ± SE (n = 3–4 independent biological replicates). Values not connected by the same letter are significantly different (Student’s t-test, p < 0.05).

### Chl *a* and Carotenoid Contents in AtLQY1-Expressing *Synechocystis*

At a growth light of 25 µmol photons m^-2^ s^-1^, AtLQY1-expressing *Synechocystis* and the empty-vector control had a similar Chl *a* content (**Table 1**). As the growth light intensity increased from 25 to 50 µmol photons m^-2^ s^-1^, the Chl *a* content in the empty-vector control decreased significantly (20%), while the Chl *a* content in AtLQY1-expressing *Synechocystis* only slightly decreased (8%,). Consequently, the Chl *a* content in AtLQY1-expressing *Synechocystis* was slightly (11%) higher than that in the empty-vector control, at 50 µmol photons m^-2^ s^-1^ (**Table 1**).

At 25 µmol photons m^-2^ s^-1^, AtLQY1-expressing *Synechocystis* and the empty-vector control had a similar carotenoid content (**Table 1**). As the growth light intensity increased from 25 to 50 µmol photons m^-2^ s^-1^, the carotenoid content in the empty-vector control did not change. The carotenoid content in AtLQY1-expressing *Synechocystis* grown at 50 µmol photons m^-2^ s^-1^ increased slightly (19%) (**Table 1**). Carotenoids have been shown to protect photosynthetic apparatus from photo-oxidation, especially under elevated light intensities (Steiger et al., 1999).

### Phycobilisome Pigment Contents in AtLQY1-Expressing *Synechocystis*

At a growth light of 25 µmol photons m^-2^ s^-1^, the APCB pigment content in AtLQY1-expressing *Synechocystis* was significantly (25%) lower than that in the empty-vector control (**Table 1**). As the growth light intensity increased from 25 to 50 µmol photons m^-2^ s^-1^, the APCB level in the empty-vector control and AtLQY1-expressing *Synechocystis* showed a 58% and 41% decrease, respectively (**Table 1**). Therefore, at 50 µmol photons m^-2^ s^-1^, the APCB content in AtLQY1-expressing *Synechocystis* was similar to that in the empty-vector control (**Table 1**).

At 25 µmol photons m^-2^ s^-1^, the PCB pigment content in AtLQY1-expressing *Synechocystis* was significantly (26%) lower than that in the empty-vector control (**Table 1**). As the growth light intensity increased from 25 to 50 µmol photons m^-2^ s^-1^, the PCB level in the empty-vector control decreased slightly (13%) while the PCB level in AtLQY1-expressing *Synechocystis* increased slightly (15%). Thus, the PCB content in the empty-vector control and AtLQY1-expressing *Synechocystis* was comparable at 50 µmol photons m^-2^ s^-1^ (**Table 1**).

Compared to APCB and PCB, the level of PEB pigment in *Synechocystis* was much lower (Hsieh et al., 2014). At 25 µmol photons m^-2^ s^-1^, the PEB content in AtLQY1-expressing *Synechocystis* was slightly (23%) lower than that in the empty-vector control (**Table 1**). As the growth light intensity increased from 25 to 50 µmol photons m^-2^ s^-1^, the PE level in both AtLQY1-expressing *Synechocystis* and the empty-vector control became negligible (**Table 1**). These light-dependent changes in *Synechocystis* pigment contents (APCB, PCB, and PEB) have been observed in previous studies (Hsieh et al., 2014).

The PCB/APCB ratio is a good indicator of the rod lengths of phycobilisomes (Chenu et al., 2017). *Synechocystis* may change the PCB/APCB ratio as an adaption response to different light intensities (Chenu et al., 2017). Therefore, we calculated the PCB/APCB ratio for *Synechocystis* cultures grown at different light intensities (**Table 1**). AtLQY1-expressing *Synechocystis* and the empty-vector control had a PCB/APCB ratio of ∼2.70 when grown at 25 µmol photons m^-2^ s^-1^ (**Table 1**). As the growth light intensity increased from 25 to 50 µmol photons m^-2^ s^-1^, the PCB/APCB ratio in the empty-vector control and AtLQY1-expressing *Synechocystis* increased by 111% and 97%, respectively (**Table 1**). Consequently, the PCB/APCB ratio in AtLQY1-expressing *Synechocystis* was slightly (7%) lower than that in the empty-vector control at 50 µmol photons m^-2^ s^-1^ (**Table 1**). This suggests that AtLQY1-expressing *Synechocystis* may have slightly shorter phycobilisome rods than the empty-vector control, at 50 µmol photons m^-2^ s^-1^.

### AtLQY1-Expressing *Synechocystis* Had Significantly Higher *F* *_v_* */F* *_m_* Than the Empty-Vector Control at 50 µmol Photons m^-2^ s^-1^

To analyze whether expressing AtLQY1 in *Synechocystis* is beneficial to PSII, we determined *F* *_v_* */F* *_m_* of dark-adapted cultures. At a growth light of 25 µmol photons m^-2^ s^-1^, AtLQY1-expressing *Synechocystis* and the empty-vector control had similar *F* *_v_* */F* *_m_*: 0.26 (**Table 1**). In cyanobacteria, the *F* *_v_* */F* *_m_* value is typically ∼0.3 instead of ∼0.8 in higher plants. One reason for this difference is that cyanobacteria have very high PSI:PSII ratios (e.g., 2:1 to 5:1), while higher plants have a PSI:PSII ratio close to 1:1 (Shen et al., 1993; Murakami et al., 1997; Luimstra et al., 2018). Another reason is that fluorescence from phycobilisomes also contributes to *F* *_o_* (Campbell et al., 1998). However, the fluorescence contribution from phycobilisomes to *F* *_o_* is fairly constant during a measurement; thus, *F* *_v_* */F* *_m_* is still a useful relative measure of PSII maximum photochemical efficiency in cyanobacteria, if neither the PSI:PSII ratio nor the phycobilisome amount is changed (Campbell et al., 1998). As the growth light intensity increased from 25 to 50 µmol photons m^-2^ s^-1^, *F* *_v_* */F* *_m_* in AtLQY1-expressing *Synechocystis* displayed a significant increase (33%). Consequently, *F* *_v_* */F* *_m_* in AtLQY1-expressing *Synechocystis* was significantly (22%) higher than that in the empty-vector control, at a growth light of 50 µmol photons m^-2^ s^-1^ (**Table 1**). This observation suggests that AtLQY1 expression in *Synechocystis* is beneficial to PSII at 50 µmol photons m^-2^ s^-1^.

A high *F* *_v_* */F* *_m_* [(*F* *_v_* */F* *_m_* = (*F* *_m_* – *F* *_o_*)/*F* *_m_* = 1 – *F* *_o_*/*F* *_m_*)] value could be the result of a low *F* *_o_* or a high *F* *_v_* value. To identify the causal parameter(s) for increased *F* *_v_* */F* *_m_* in AtLQY1-expressing *Synechocystis*, we determined *F* *_o_*, *F* *_m_*, and *F* *_v_* of dark-adapted cultures. At a growth light of 25 µmol photons m^-2^ s^-1^, the *F* *_o_*, *F* *_m_*, and *F* *_v_* values in dark-adapted AtLQY1-expressing *Synechocystis* were approximately 15-17% higher than those in the dark-adapted empty-vector control (**Table 1**). Due to the coordinated increases in these three parameters, *F* *_v_* */F* *_m_* in AtLQY1-expressing *Synechocystis* was similar to that in the empty-vector control, at 25 µmol photons m^-2^ s^-1^ (**Table 1**). At a growth light of 50 µmol photons m^-2^ s^-1^, the *F* *_o_*, *F* *_m_*, and *F* *_v_* values in dark-adapted AtLQY1-expressing *Synechocystis* were 55%, 70%, and 109% higher than those in the empty-vector control, respectively (**Table 1**). This suggests that the high *F* *_v_* */F* *_m_* value in AtLQY1-expressing *Synechocystis* grown at 50 µmol photons m^-2^ s^-1^ is mostly the effect of high *F* *_v_*. In cyanobacteria as well as land plants, *F* *_v_* arises essentially from PSII; thus a higher *F* *_v_* value is indicative of a high ability of PSII to perform primary photochemistry (Campbell et al., 1998; Baker et al., 2007).

**Figure 2:**
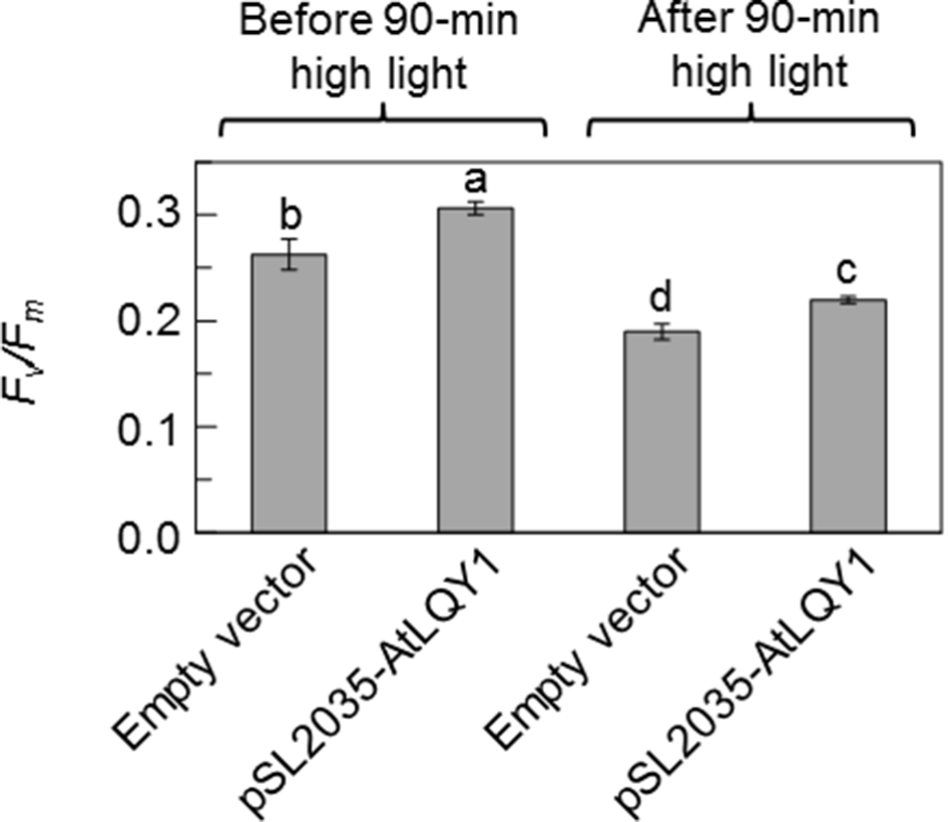
*F* *_v_* */F* *_m_* values before and after a 90-min moderately high light treatment at 250 µmol photons m^-2^ s^-1^. Prior to high light the treatment, *Synechocystis* cultures were grown at 50 µmol photons m^-2^ s^-1^. Data are presented as mean ± SE (n = 4 independent biological replicates). Values not connected by the same lowercase letter are significantly different (Student’s *t*-test, p < 0.05).

We also subjected *Synechocystis* cultures grown at 50 µmol photons m^-2^ s^-1^ to a 90-min moderately high light treatment at 250 µmol photons m^-2^ s^-1^ and determined *F* *_v_* */F* *_m_* before and after the high light treatment (**Figure 2**, **Supplementary Table S2**). We found that *F* *_v_* */F* *_m_* in AtLQY1-expressing *Synechocystis* was significantly (∼16%) higher than that in the empty-vector control before and after the high light treatment at 250 µmol photons m^-2^ s^-1^. This suggests that AtLQY1-expressing *Synechocystis* outperforms the empty-vector control at higher growth light intensities.

### AtLQY1-Expressing *Synechocystis* Grown at 50 µmol Photons m^-2^ s^-1^ Had Significantly Higher *Φ* *_PSII_* and *ETR* *_PSII_* Than the Empty-Vector Control Under High Measuring Light Intensities

**Figure 3:**
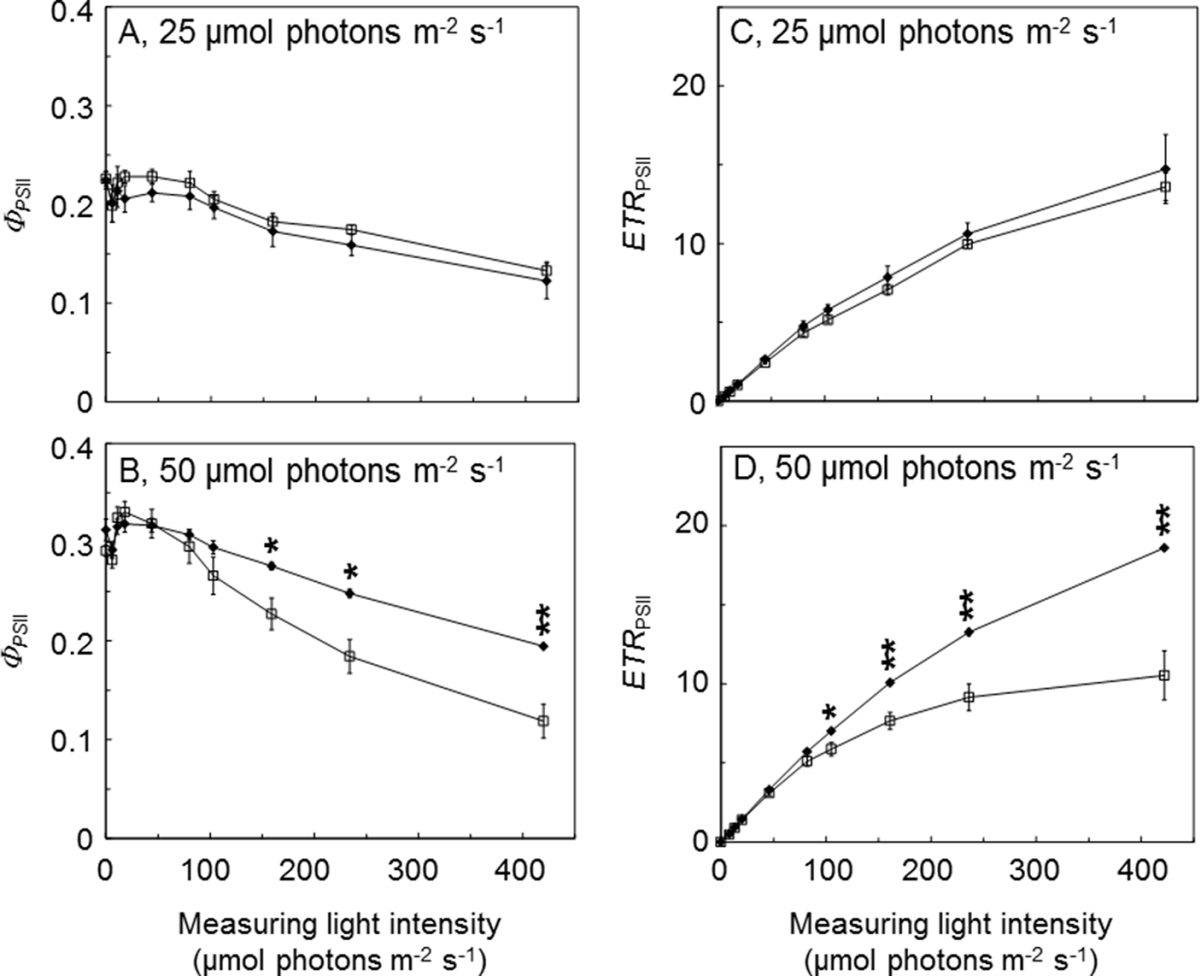
Light response curves of PSII operating efficiency and electron transport rate. **(A**, **B)** Light response curves of PSII operating efficiency (Φ*_PSII_*) in the empty-vector control (white squares) and AtLQY1-expressing *Synechocystis* (black diamonds) grown at 25 **(A)** and 50 **(B)** µmol photons m^-2^ s^-1^. **(C**, **D)** Light response curves of PSII electron transport rate (*ETR*_PSII_) in the empty-vector control and AtLQY1-expressing *Synechocystis* grown at 25 **(C)** and 50 **(D)** µmol photons m^-2^ s^-1^. Initial cultures were started at an optical density of 0.05 at 730 nm. The light response curves were determined on mid-log phase cultures after a 5-min dark adaption. During the measurement, cultures were exposed for 30 s at a wide range of light intensities (0, 8, 13, 20, 46, 82, 105, 161, 236, and 422 µmol photons m^-2^ s^-1^). Data are presented as mean ± SE (n = 4 independent biological replicates). Asterisks indicate significant differences between AtLQY1-expressing *Synechocystis* and the empty-vector control (Student’s *t*-test; *, *p* < 0.05; **, *p* < 0.01).

To further investigate the benefits of AtLQY1 expression in *Synechocystis*, we determined the light response curves of *Φ* *_PSII_* and *ETR* *_PSII_* (**Figure 3**; **Supplementary Table S2**). AtLQY1-expressing *Synechocystis* and the empty-vector control grown at 25 µmol photons m^-2^ s^-1^ had no statistically significant difference in *Φ* *_PSII_* or *ETR* *_PSII_*, under all the measuring light intensities tested (**Figures 3A, C**). When grown at 50 µmol photons m^-2^ s^-1^, AtLQY1-expressing *Synechocystis* and the empty-vector control had statistically similar *Φ* *_PSII_* and *ETR* *_PSII_* under low measuring light intensities, such as 8, 13, 20, and 46 µmol photons m^-2^ s^-1^ (**Figures 3B, D**). The initial slope of *ETR*_PSII_ light response curves is a measure of PSII antenna size, i.e., light harvesting capacity (Sauer et al., 2001; Yamazaki et al., 2005). The identical initial slope of the two *ETR* *_PSII_* light response curves (**Figure 3D**) suggests that PSII antenna size in AtLQY1-expressing *Synechocystis* is similar to that in the empty-vector control. AtLQY1-expressing *Synechocystis* started to show slight but statistically insignificant advantages at the measuring light intensities of 82 µmol photons m^-2^ s^-1^ (**Figures 3B, D**). Under high measuring light intensities, i.e., 161, 236, and 422 µmol photons m^-2^ s^-1^, AtLQY1-expressing *Synechocystis* displayed significantly higher *Φ* *_PSII_* and *ETR* *_PSII_* than the empty-vector control: *Φ* *_PSII_* in AtLQY1-expressing *Synechocystis* was 21%, 35%, and 64% higher than that in the empty-vector control whereas *ETR* *_PSII_* in AtLQY1-expressing *Synechocystis* was 32%, 45%, and 77% higher than that in the empty-vector control (**Figures 3B, D**). *ETR* *_PSII_* in the empty-vector control plateaued at the measuring light of 236 µmol photons m^-2^ s^-1^ while *ETR*_PSII_ in AtLQY1-expressing *Synechocystis* continued to increase as the measuring light intensity increased. This indicates that AtLQY1-expressing *Synechocystis* devotes a higher percentage of excitation energy into photochemistry than the empty-vector control, under high light intensities.

### AtLQY1-Expressing *Synechocystis* Had a Significantly Lower *NPQ* Value Than the Empty-Vector Control at 50 µmol Photons m^-2^ s^-1^

In photosynthetic organisms, excess excitation energy can be coped with by mechanisms such as thermal dissipation (Campbell and Oquist, 1996; Campbell et al., 1998; Müller et al., 2001; Gorbunov et al., 2011; Jahns and Holzwarth, 2012; Kress and Jahns, 2017). Although cyanobacteria lack the xanthophyll-cycle-mediated energy-dependent quenching, they can regulate excitation energy via state transitions and NPQ, which is mediated by the orange carotenoid protein (Campbell and Oquist, 1996; Campbell et al., 1998; Gorbunov et al., 2011). Therefore, we measured NPQ in AtLQY1-expressing *Synechocystis* and the empty-vector control. At a growth light of 25 µmol photons m^-2^ s^-1^, AtLQY1-expressing *Synechocystis* and the empty-vector control had a similar *NPQ* value (**Table 1**). Both AtLQY1-expressing *Synechocystis* and the empty-vector control displayed reduced *NPQ* values as the growth light intensity increased from 25 to 50 µmol photons m^-2^ s^-1^. This concave dependence of *NPQ* on actinic light intensities has been previously reported in *Synechocystis* and other cyanobacterial species (Campbell and Oquist, 1996; Misumi et al., 2016; Ogawa and Sonoike, 2016; Misumi and Sonoike, 2017). As the growth light intensity increased from 25 to 50 µmol photons m^-2^ s^-1^, *NPQ* in the empty-vector control showed a 19% reduction whereas AtLQY1-expressing *Synechocystis* displayed a 45% reduction. As a result, *NPQ* in AtLQY1-expressing *Synechocystis* was significantly (32%) lower than that in the empty-vector control, at a growth light of 50 µmol photons m^-2^ s^-1^ (**Table 1**). This observation suggests that AtLQY1 expression in *Synechocystis* may reduce NPQ at certain growth light conditions, such as 50 µmol photons m^-2^ s^-1^.

### AtLQY1-Expressing *Synechocystis* Had a Significantly Lower Amount of ROS Than the Empty-Vector Control at 50 µmol Photons m^-2^ s^-1^

**Figure 4:**
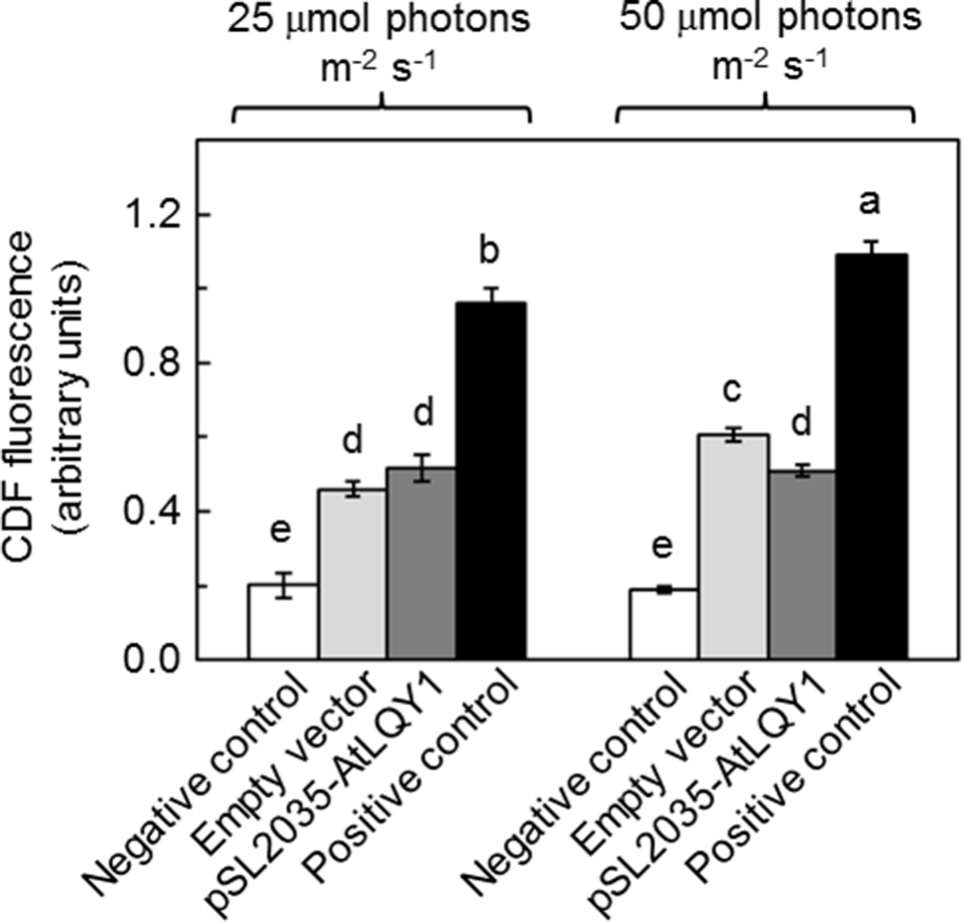
Measurement of ROS accumulation in *Synechocystis* cultures grown at 25 and 50 µmol photons m^-2^ s^-1^. Data are presented as mean ± SE (n = 4 independent biological replicates). Values not connected by the same lowercase letter are significantly different (Student’s *t*-test, *p* < 0.05).

The light response curves of *ETR* *_PSII_* suggested that AtLQY1-expressing *Synechocystis* allocates a higher ratio of excitation energy into photochemistry than the empty-vector control, under higher light intensities. It is commonly known that there is more oxidative damage to photosynthetic organisms at higher light intensities. This prompted us to measure the amount of ROS in *Synechocystis* cultures at both growth light intensities (**Figure 4**, **Supplementary Table S2**). At a growth light of 25 µmol photons m^-2^ s^-1^, AtLQY1-expressing *Synechocystis* and the empty-vector control had a similar total ROS content (**Figure 4**). As the growth light intensity increased from 25 to 50 µmol photons m^-2^ s^-1^, the amount of ROS in the empty-vector control increased significantly (32%) whereas the ROS content in AtLQY1-expressing *Synechocystis* did not change (**Figure 4**). Consequently, at a growth light of 50 µmol photons m^-2^ s^-1^, the ROS level in AtLQY1-expressing *Synechocystis* was 16% lower than that in the empty-vector control (**Figure 4**). This observation suggests that AtLQY1 expression in *Synechocystis* may reduce ROS accumulation at certain growth light conditions, such as 50 µmol photons m^-2^ s^-1^.

### The Amounts of Cysteine-Containing Proteins in AtLQY1-Expressing *Synechocystis*

**Figure 5:**
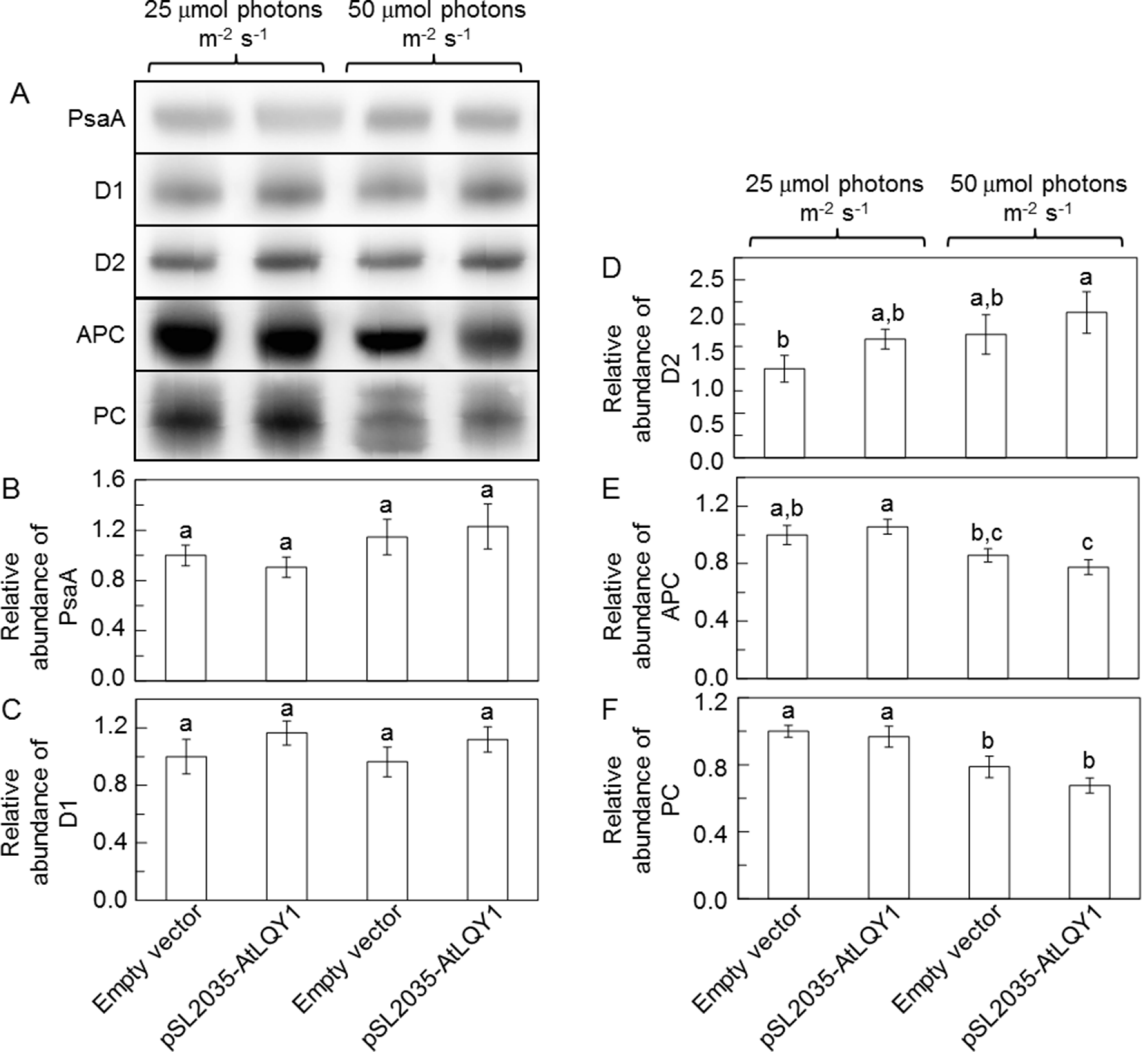
Immunoblot analysis of protein abundances in *Synechocystis* cultures grown at 25 and 50 µmol photons m^-2^ s^-1^. **(A)** Representative immunoblots of PSI core protein PsaA, PSII core proteins D1 and D2, and phycobilisome proteins APC and PC. Total protein samples were loaded on an equal culture OD_730_ basis. **(B**−**F)** Relative abundances of PsaA, D1, D2, APC, and PC proteins. Data are presented as mean ± SE (n = 3 independent biological replicates). Values not connected by the same lowercase letter are significantly different (Student’s *t*-test, p < 0.05).

Recombinant AtLQY1 protein displayed thiol/disulfide-modulating activity towards thiol/disulfide-containing protein substrates (Lu et al., 2011). Therefore, we determined the amounts of representative cysteine-containing PSI and PSII core proteins in AtLQY1-expressing *Synechocystis* and the empty-vector control (**Figure 5**, **Supplementary Table S2**). PSI core protein PsaA in *Synechocystis* contains four cysteine residues. The abundance of PsaA in AtLQY1-expressing *Synechocystis* was statistically similar to that in the empty-vector control at both growth light intensities (**Figure 5B**). PSII core protein D1 in *Synechocystis* contains four cysteine residues. The abundance of D1 in AtLQY1-expressing *Synechocystis* was slightly (16%) higher than that in the empty-vector control under both growth light conditions (**Figure 5C**). This observation is consistent with the proposed role of AtLQY1 in the PSII repair and reassembly cycle. PSII core protein D2 in *Synechocystis* contains two cysteine residues. Interestingly, the amount of D2 was slightly higher than that in the empty-vector control under both growth light intensities: 33% higher at 25 µmol photons m^-2^ s^-1^ and 18% higher at 50 µmol photons m^-2^ s^-1^ (**Figure 5D**).

Phycobilisome proteins APC, PC, and PE also contain conserved cysteine residues, to which phycobilisome chromophores APCB, PCB, and PEB, are covalently attached (Zhao et al., 2006). Therefore, we determined the amounts of APC and PC proteins (**Figure 5**, **Supplementary Table S2**). The APC content in the empty-vector control and AtLQY1-expressing *Synechocystis* was similar at 25 µmol photons m^-2^ s^-1^ (**Figure 5E**). As the growth light intensity increased from 25 to 50 µmol photons m^-2^ s^-1^, the APC level in the empty-vector control and AtLQY1-expressing *Synechocystis* reduced by 14% and 27%, respectively (**Figure 5E**). Consequently, the APC amount in AtLQY1-expressing *Synechocystis* was slightly (10%) lower than that in the empty-vector control at 50 µmol photons m^-2^ s^-1^ (**Figure 5E**). The PC content displayed a similar pattern as APC (**Figure 5F**).

### Thylakoid Structures of AtLQY1-Expressing *Synechocystis*

**Figure 6:**
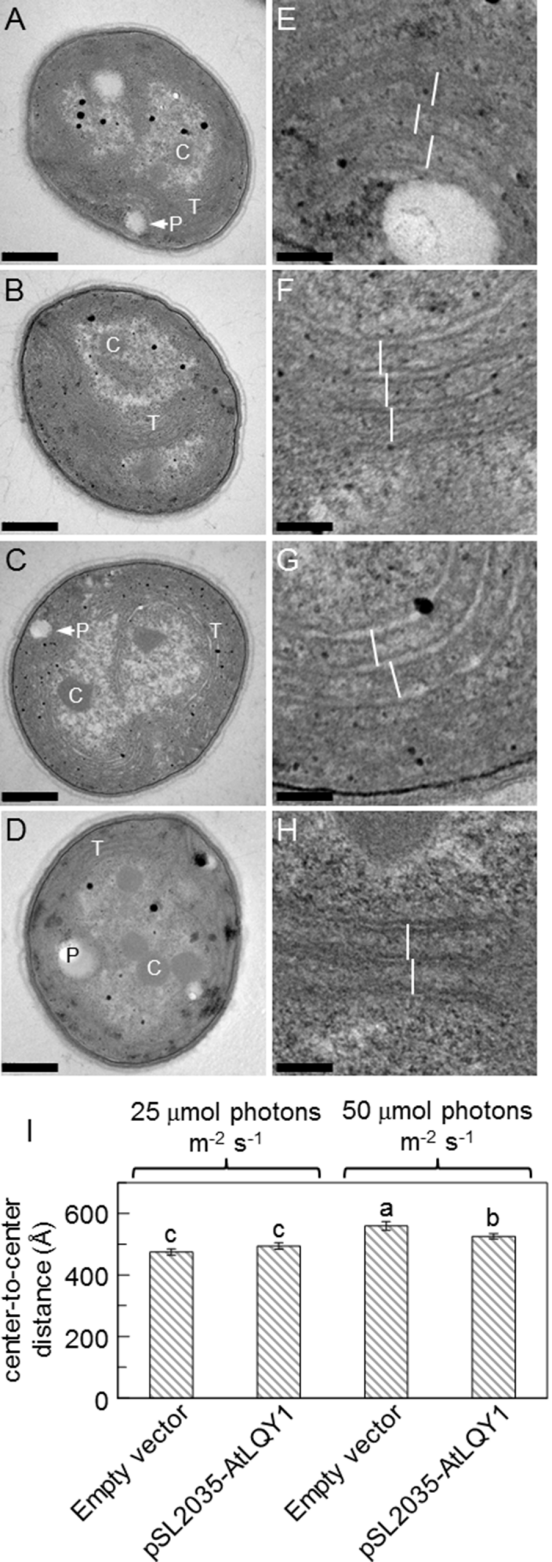
Transmission electron micrographs of the empty-vector control and AtLQY1-expressing *Synechocystis.* **(A**–**D)** Whole cell images of the empty-vector control **(A, C)** and AtLQY1-expressing *Synechocystis* **(B**, **D)** grown at 25 **(A**, **B)** and 50 **(C**, **D)** µmol photons m^-2^ s^-1^. C, carboxysomes; P, polyphosphate bodies or holes left by polyphosphate bodies; T, thylakoid membranes. Scale bars in A-D: 400 nm. **(E**–**H)** Enlargements of the empty-vector control **(E**, **G)** and AtLQY1-expressing *Synechocystis* **(F**, **H)** grown at 25 **(E**, **F)** and 50 **(G**, **H)** µmol photons m^-2^ s^-1^. White lines depict thylakoid membrane spacing distances. Scale bars in **E**–**H**: 80 nm. **(I)** Thylakoid membrane spacing distances. Data are presented as mean ± SE (n = 30 independent biological replicates). Values not connected by the same lowercase letter are significantly different (Student’s *t*-test, *p* < 0.05)

We analyzed thylakoid structures of AtLQY1-expressing *Synechocystis* and the empty-vector control with TEM (**Figure 6**). The overall cell morphology of AtLQY1-expressing *Synechocystis* and the empty-vector control was quite similar, with visible thylakoid membranes and carboxysomes, at both growth light intensities (**Figures 6A–D**). It was previously found that in cyanobacteria, thylakoid membrane spacing distance depends on the presence and size of extrinsic phycobilisomes and that light induces the expansion of thylakoid membrane spacing distance (Olive et al., 1997; Nagy et al., 2011; Collins et al., 2012; Liberton et al., 2013; Stingaciu et al., 2016; Majumder et al., 2017). Therefore, we measured thylakoid membrane spacing distances in TEM images of *Synechocystis* grown at different light intensities (**Figures 6E–H**). The empty-vector control grown at 25 µmol photons m^-2^ s^-1^ had an average thylakoid membrane spacing distance of 475 ± 10 Å (i.e., 47.5 ± 1.0 nm) (**Figure 6I**, **Supplementary Table S2**), which is comparable to wild-type *Synechocystis* grown under the same conditions (white light at 25 µmol photons m^-2^ s^-1^) (Liberton et al., 2013). AtLQY1-expressing *Synechocystis* grown at 25 µmol photons m^-2^ s^-1^ had an average thylakoid membrane spacing distance of 494 ± 11 Å, statistically similar to the empty-vector control (**Figure 6I**). As the growth light intensity increased from 25 to 50 µmol photons m^-2^ s^-1^, the thylakoid membrane spacing distance in the empty-vector control and AtLQY1-expressing *Synechocystis* increased by 18% and 6% respectively, both of which are statistically significant (**Figure 6I**). Consequently, at a growth light intensity of 50 µmol photons m^-2^ s^-1^, the thylakoid membrane spacing distance in AtLQY1-expressing *Synechocystis* was significantly (6%) lower than that in the empty-vector control (**Figure 6I**). This observation suggests that AtLQY1 expression in *Synechocystis* may reduce light-induced expansion of thylakoid membrane spacing distance at certain growth light conditions, such as 50 µmol photons m^-2^ s^-1^. This is consistent with the slightly lower PCB/APCB ratio, which is indicative of phycobilisome rod lengths, in AtLQY1-expressing *Synechocystis* grown at 50 µmol photons m^-2^ s^-1^ (**Table 1**).

## Discussion

Measurements of different photosynthetic parameters consistently showed that AtLQY1 expression improves PSII photochemical efficiency in *Synechocystis* cells grown at 50 µmol photons m^-2^ s^-1^. In higher plants, *F* *_v_* */F* *_m_* is well established as the indicator of the maximum quantum efficiency of PSII photochemistry (Campbell et al., 1998; Baker et al., 2007). In cyanobacteria, *F* *_v_* */F* *_m_* is still a useful relative measure of the maximum photochemical efficiency of PSII (Campbell et al., 1998; Sauer et al., 2001). When grown at 50 µmol photons m^-2^ s^-1^, AtLQY1-expressing *Synechocystis* had significantly (22%) higher *F* *_v_* */F* *_m_* than the empty-vector control (**Table 1**), suggesting that introducing AtLQY1 into *Synechocystis* may increase the maximum photochemical efficiency of PSII. At 50 µmol photons m^-2^ s^-1^, the amount of PSII core protein D1 increased in AtLQY1-expressing *Synechocystis* by ∼16% (**Figure 5C**), while the amount of PSI core protein PsaA remained relatively unchanged (**Figure 5B**). Therefore, the increase in *F* *_v_* */F* *_m_* most likely comes from the increased amount of PSII. Further analysis of chlorophyll fluorescence parameters showed that AtLQY1-expressing *Synechocystis* grown at 50 µmol photons m^-2^ s^-1^ also had higher *F* *_v_* than the empty-vector control. In both cyanobacteria and land plants, *F* *_v_* comes mostly from PSII (Campbell et al., 1998; Baker et al., 2007); thus, the increase of PSII relative to PSI can explain the increase of *F* *_v_* */F* *_m_* and also of *F* *_v_*. In oxygenic photosynthetic organisms such as plants, algae, and cyanobacteria, *Φ* *_PSII_* and *ETR* *_PSII_* estimate the efficiency of PSII photochemistry and the rate of non-cyclic electron transport through PSII at given light intensities (Sauer et al., 2001; Baker et al., 2007; Barthel et al., 2013). Interestingly, AtLQY1-expressing *Synechocystis* grown at 50 µmol photons m^-2^ s^-1^ also had higher *Φ* *_PSII_* and *ETR* *_PSII_* than the empty-vector control, at high measuring light intensities (e.g., 161-422 µmol photons m^-2^ s^-1^, **Figure 3**). This suggests that AtLQY1 expression in *Synechocystis* may improve PSII operating efficiency and electron transport rate under higher light intensities. AtLQY1-expressing *Synechocystis* grown at 50 µmol photons m^-2^ s^-1^ was also found to have lower *NPQ* and ROS levels than the corresponding empty-vector control (**Table 1**, **Figure 4**). This indicates that AtLQY1 expression in *Synechocystis* may reduce NPQ and ROS accumulation at certain light intensities, such as 50 µmol photons m^-2^ s^-1^.

Many phenotypic advantages of AtLQY1-expressing *Synechocystis* grown at 50 µmol photons m^-2^ s^-1^ were not seen when the *Synechocystis* cells were grown at 25 µmol photons m^-2^ s^-1^. According to Trautmann et al. (2016), the growth of S*ynechocysti*s transits from light-limited to light-saturated at around 46 µmol photons m^-2^ s^-1^. When the light intensity is below 46 µmol photons m^-2^ s^-1^, S*ynechocysti*s growth is light-limited and the growth rate is proportional directly to the light intensity (Trautmann et al., 2016). When the light intensity exceeds 46 µmol photons m^-2^ s^-1^, S*ynechocysti*s growth transits to light-saturated (Trautmann et al., 2016). Consistent with these findings, AtLQY1-expressing *Synechocystis* grown at 50 µmol photons m^-2^ s^-1^ displayed advantages in PSII operating efficiency and electron transport rate (**Figures 3B, D**). Taken together, although AtLQY1 expression does not improve the efficiency of PSII photochemistry of S*ynechocysti*s under light-limited conditions, it significantly improves PSII photochemical efficiency, when the light intensity exceeds the threshold value of 46 µmol photons m^-2^ s^-1^.

As discussed above, AtLQY1-expressing *Synechocystis* had significantly higher *F* *_v_*, *F* *_v_* */F* *_m_*, Φ*_PSII_*, and *ETR*_PSII_, lower *NPQ* and ROS levels, and a shorter thylakoid membrane spacing distance than the empty-vector control, when grown at 50 µmol photons m^-2^ s^-1^. It is possible that AtLQY1 exerts these effects by participating in the folding, disassembly, and assembly of cysteine-containing PSII subunits, and reducing ROS accumulation. Consistent with this possibility, AtLQY1-expressing *Synechocystis* had a slightly higher amount of cysteine-containing PSII core protein D1 as well as an opposite phenotype of loss-of-function Arabidopsis mutants of AtLQY1, which was proposed to assist in the repair and reassembly cycle of PSII and redox homeostasis in Arabidopsis (Lu, 2011; Lu et al., 2011). It’s possible that AtLQY1 exerts these effects by reducing OCP-mediated NPQ, potentially via modulating the redox status of thiol-containing cysteine residues in OCP homodimers. In line with this possibility, AtLQY1-expressing *Synechocystis* had a lower *NPQ* value than the empty-vector control, when grown at 50 µmol photons m^-2^ s^-1^. AtLQY1 may also influence phycobilisome assembly, and/or association between phycobilisome proteins and their respective chromophores. Consistent with this possibility, AtLQY1-expressing *Synechocystis* had a shorter thylakoid membrane spacing distance and a slightly lower PCB/APCB ratio (indicative of shorter phycobilisome rod lengths) than the empty-vector control, when grown at 50 µmol photons m^-2^ s^-1^. Further studies are needed to unveil the mechanisms behind the observed phenotypes, in AtLQY1-expressing *Synechocystis*.

Photochemistry, NPQ, and photoinhibition are competing processes in photosynthetic organisms (Arsalane et al., 1994; Horton et al., 1996; Müller et al., 2001; Lambrev et al., 2012; Berteotti et al., 2016; Pathak et al., 2019). NPQ evolved as a photoprotective mechanism to minimize photodamage and photoinhibition. Therefore, reductions in NPQ are often associated with increases in ROS accumulation. For example, NPQ in green algae is mediated by LHC-like proteins known as LHCSRs (Bonente et al., 2011; Berteotti et al., 2016; Correa-Galvis et al., 2016; Tibiletti et al., 2016; Perozeni et al., 2019). Disruption of all three *lhcsr* genes resulted in enhanced ROS production (Berteotti et al., 2016; Perozeni et al., 2019). However, enhanced ROS accumulation can also be accompanied by increases in NPQ. For example, loss-of-function *Atlqy1* and *hypersensitive to high light1* (*hhl1*) Arabidopsis mutants displayed simultaneous increases in NPQ and ROS under high light conditions (Lu, 2011; Lu et al., 2011; Jin et al., 2014). Therefore, it is conceivable to observe simultaneous decreases in NPQ and ROS in AtLQY1-expressing *Synechocystis.* Furthermore, because NPQ reduces photodamage and photoinhibition at the cost of reduced photosynthetic efficiency, down regulation of NPQ was recently found to be a suitable strategy to improve photosynthetic efficiency in land plants and green algae (Berteotti et al., 2016; Kromdijk et al., 2016; Perozeni et al., 2019).

*Synechocystis* has a set of endogenous chloroplastic thiol/disulfide-modulating proteins (Nixon et al., 2010; Mulo et al., 2012; Nickelsen and Rengstl, 2013; Lu, 2016). Loss-of-function mutations in genes encoding these chloroplastic thiol/disulfide-modulating proteins were found to have pleiotropic effects, e.g., reductions in *F* *_v_* */F* *_m_*, increases in NPQ, increased photoinhibition, enhanced ROS accumulation, and deficiencies in the assembly and stability of photosynthetic apparatus (Karamoko et al., 2011; Calderon et al., 2013; Wang et al., 2013). One example is thylakoid membrane-anchored LTO1 (encoded by slr0565 in *Synechocystis*). As mentioned in the introduction, *Synechocystis* and *Arabidopsis* homologs of this protein were reported to catalyze disulfide bond formation in lumenal and lumen-exposed proteins, such as FKBP13, PsbO1, and PsbO2 (Singh et al., 2008a; Furt et al., 2010; Li et al., 2010; Feng et al., 2011; Karamoko et al., 2011; Lu et al., 2013). The *lto1* mutant showed reduced *F* *_v_* */F* *_m_*, increased NPQ, increased photoinhibition, and deficient PSII assembly (Karamoko et al., 2011). A second example is thylakoid membrane-anchored rubredoxin 1 (RBD1, encoded by slr2033 in *Synechocystis*). *Synechocystis*, *Chlamodonas reinhardtii*, and *Arabidopsis* homologs of this protein were found to be necessary for PSII activity and were therefore proposed to play a role in promoting PSII assembly and stability (Calderon et al., 2013). The *rbd1* mutant displayed very low *F* *_v_* */F* *_m_* and severely impaired PSII accumulation (Calderon et al., 2013). A third example is chloroplast stromal m-type thioredoxin (trx-M, encoded by slr0623 in *Synechocystis*). Although the role of *Synechocystis* trx-M in PSII assembly and repair has not been reported, its Arabidopsis homologs (TRX-M1, TRX-M2, and TRX-M4) were found to participate in the assembly of CP47 into PSII (Cain et al., 2009; Wang et al., 2013). Disruption of all three *trx-m* genes resulted in reduced *F* *_v_* */F* *_m_*, increased NPQ, enhanced ROS production, and reduced PSII stability (Wang et al., 2013). Therefore, it is conceivable to observe that AtLQY1 expression in *Synechocystis* had pleiotropic effects (e.g., increased *F* *_v_* */F* *_m_*, *Φ* *_PSII_*, and *ETR* *_PSII_*, reduced NPQ, and decreased ROS content).

Unlike LQY1, these three thiol/disulfide-modulating proteins are present ubiquitously in cyanobacteria, algae, and land plants (Lu, 2016). It is interesting that introducing AtLQY1, an Arabidopsis thylakoid membrane-anchored thiol/disulfide-modulating protein, into *Synechocystis*, which contains three endogenous chloroplastic thiol/disulfide-modulating proteins, is still beneficial to the organism. One possibility is that these four proteins target different thiol/disulfide-containing proteins, depending on the locations of these thiol/disulfide-modulating proteins in the chloroplast. For instance, LTO1 may target lumenal and lumen-exposed thiol/disulfide–containing proteins (Singh et al., 2008a; Furt et al., 2010; Li et al., 2010; Feng et al., 2011; Karamoko et al., 2011; Lu et al., 2013); Trx-M may target soluble thiol/disulfide–containing proteins in the chloroplast stroma (Cain et al., 2009; Wang et al., 2013). In Arabidopsis, LQY1 is a thylakoid membrane protein with its N-terminal transmembrane domain anchored in thylakoid membranes and its C-terminal zinc-finger domain in the stroma (Lu et al., 2011). Therefore, LQY1 has the potential to target thiol/disulfide–containing proteins in thylakoid membranes and stroma in land plants. Although further studies are needed to determine the subcellular location of AtLQY1 in AtLQY1-expressing *Synechocystis*, the amphipathic property of this protein suggests that AtLQY1 may target both membrane and soluble proteins.

To sum up, this study showed that introducing a land plant-derived thylakoid thiol/disulfide-modulating protein, AtLQY1, into a cyanobacterium significantly improved overall PSII efficiency of the organism. Cyanobacteria have great potential as biofuel producers, making efforts to enhance the productivity of these organisms is valuable to society (Machado and Atsumi, 2012; Nozzi et al., 2013; Varman et al., 2013; Allahverdiyeva et al., 2014; Lea-Smith and Howe, 2017; Cheregi et al., 2019). Introducing LQY1, or other land plant-derived thiol/disulfide-modulating proteins, may be a strategy to optimize cyanobacterial growth under light-saturated conditions.

## Data Availability Statement

The datasets generated for this study are available on request to the corresponding author.

## Author Contributions

RW performed the experiments, analyzed the data, and edited the manuscript. YL conceived the project, analyzed the data, and wrote and edited the manuscript.

## Funding

This work was financially supported by the U.S. National Science Foundation (grant number MCB-1244008) and the Western Michigan University Faculty Research and Creative Activities Award (grant number W2016-023).

## Conflict of Interest

The authors declare that the research was conducted in the absence of any commercial or financial relationships that could be construed as a potential conflict of interest
