# Supplementary material for: Introducing an Arabidopsis thaliana Thylakoid Thiol/Disulfide-Modulating Protein Into Synechocystis Increases the Efficiency of Photosystem II Photochemistry
Source: Front Plant Sci. 2019 Oct 16;10:1284. doi: 10.3389/fpls.2019.01284 (PMC6805722; doi:10.3389/fpls.2019.01284)
Supplement: Supplementary file 1 [file Table_1.pdf]

Supplementary Table S1. Primers used in this study		
Primer	Sequence	Use
ps1_up_fwd	CGCGGGTACCGGAACAGGACCAAGCCTT	Cloning upstream of psbA1 to inspect pLS2035 sequence
ps1_up_bwd	CGCGCAATTGGATGGTTTCTCAGATTGCAGTTGGTTTCC	Cloning upstream of psbA1 to inspect pLS2035 sequence
ps1_down_fwd	CGCGCAATTGTGCCATTGCCATAACTGCTTTCGGTTAG	Cloning downstream of psbA1 to inspect pLS2035 sequence
ps1_down_bwd	CGCGAAGCTTTGACTATCCTTTTTAGGATGGGGCAAGG	Cloning downstream of psbA1 to inspect pLS2035 sequence
Nde1_LQY1_F	GGCGACCATATGCCAGTTTCAGCTCCATCT	Cloning AtLQY1 and genotyping *Synechocystis* transformants
Hpa1_LQY1_R	GGCGAGGTTAACTTAGTCATCGTCCTTGAACTCC	Cloning AtLQY1
M13_Forward	TGTAAAACGACGGCCAGT	Sequencing inserts in pGEM-T Easy
M13_Reverse	CAGGAAACAGCTATGACC	Sequencing inserts in pGEM-T Easy
psbA1d_100_down_R	GGGTCAAACTCCATTGCCAAAAGC	Genotyping *Synechocystis* transformants
psbA1d_200_down_R	CGATAATTGCCATAGTCGTTAAAT	Genotyping *Synechocystis* transformants
